# Internal hernia of the stomach associated with colostomy after laparoscopic surgery for rectal cancer: a case report

**DOI:** 10.1186/s40792-020-00889-8

**Published:** 2020-06-03

**Authors:** Hiroki Hashida, Ryosuke Kita, Masato Kondo, Ryosuke Mizuno, Hiroyuki Kobayashi, Satoshi Kaihara

**Affiliations:** Department of Surgery, Kobe City Medical Center General Hospital, 2-1-1 Minatojima-Minamimachi, Chuo-ku, Kobe, 650-0047 Japan

**Keywords:** Colostomy, Internal hernia, Rectal cancer, Stomach

## Abstract

**Background:**

Colostomy via the intraperitoneal route is often performed during laparoscopic Hartmann’s operation or abdominoperineal resection. Internal hernia of the small intestine often occurs after colostomy. This report shows a rare case of internal hernia of the stomach associated with sigmoid colostomy after laparoscopic abdominoperineal resection for rectal cancer.

**Case presentation:**

The patient was a 79-year-old woman with a sigmoid colostomy. Computed tomography scan showed a markedly distended stomach in the space between the lifted sigmoid colon and the lateral abdominal wall. Laparoscopy revealed that the body of the stomach had passed through a hernia orifice located between the lifted sigmoid colon and the left lateral abdominal wall. The dislocated stomach was restored to its normal position, and the lateral defect was closed with the lateral peritoneum and the lifted sigmoid colon laparoscopically.

**Conclusions:**

Internal hernia associated with colostomy can lead to not only obstruction of the small intestine, but also obstruction of the stomach. We reported a successful case of the suture repair for the internal hernia of the stomach associated with colostomy.

## Background

With progressive developments in laparoscopic rectal surgery, anus-preserving procedures have become available for patients with rectal cancer [1]. However, creation of a stoma is still needed for patients with rectal malignancy who undergo Hartmann’s operation (HO) or abdominoperineal resection (APR) [2].

Major complications associated with colostomy after HO or APR are stoma stenosis, retraction, prolapse, parastomal hernia, and intestinal obstruction [3]. Major causes of postoperative intestinal obstruction are intraperitoneal adhesions and torsion. There have been few reports of intestinal obstruction resulting from internal hernia associated with colostomy (IHAC) [4]. In addition, there are several reports about parastomal hernia containing the stomach [5]. However, internal hernia of the stomach without parastomal hernia has never been described.

We herein present a rare case of internal hernia of the stomach associated with sigmoid colostomy.

## Case presentation

**Fig. 1:**
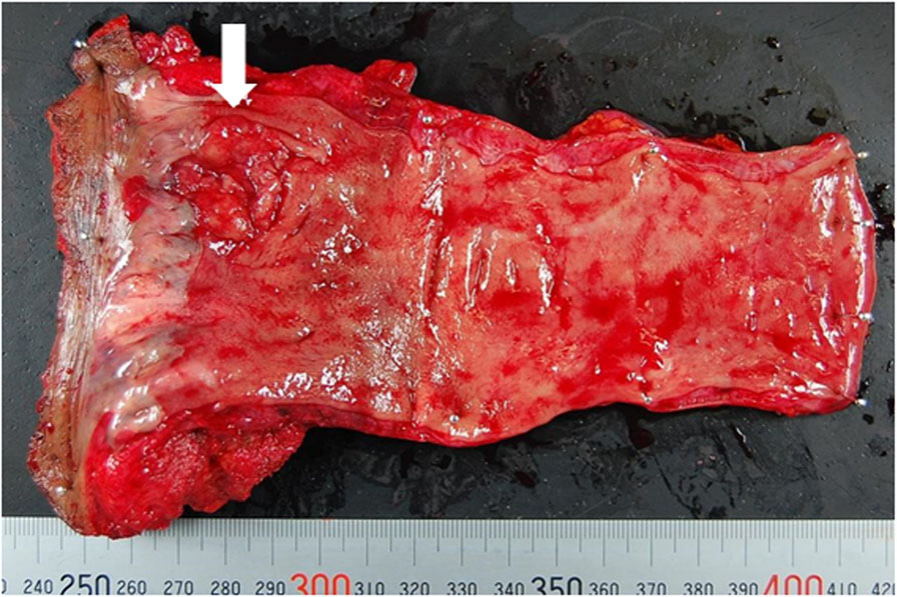
A resected specimen is shown. A tumor located on the dentate line

**Fig. 2:**
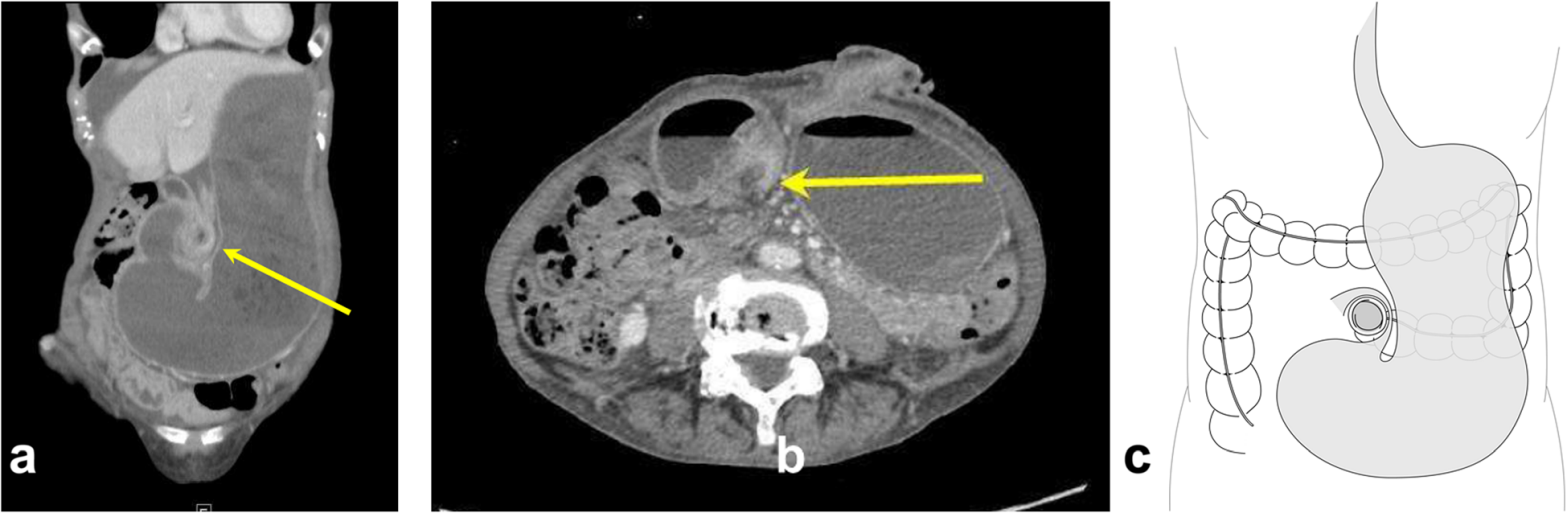
Contrast-enhanced CT shows **a** the body of the stomach and the greater omentum positioned in the lateral space between the lifted sigmoid colon and the lateral abdomen. A yellow arrow shows the lifted sigmoid colon. **b** A markedly dilated stomach and the medially displaced lifted sigmoid colon are observed (yellow arrow). **c** The schema of internal hernia of the stomach

**Fig. 3:**
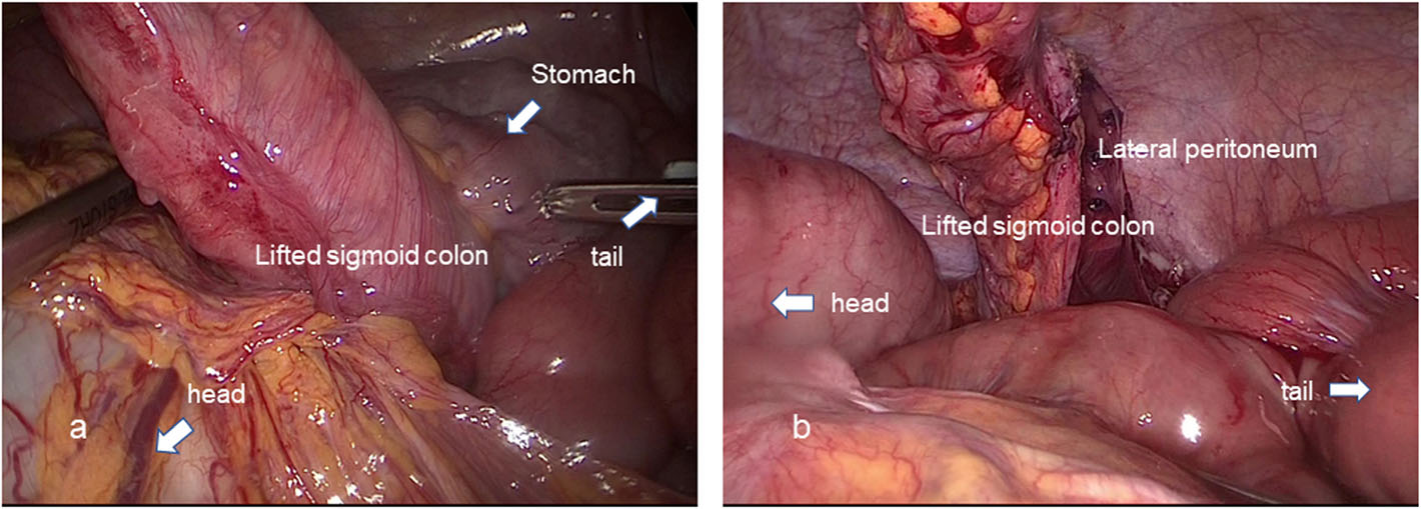
**a** Laparoscopic finding shows the part of the stomach passing through the defect between the left lateral abdominal wall and the lifted sigmoid colon. **b** The lateral defect was closed with the lateral peritoneum and the lifted sigmoid colon

**Fig. 4:**
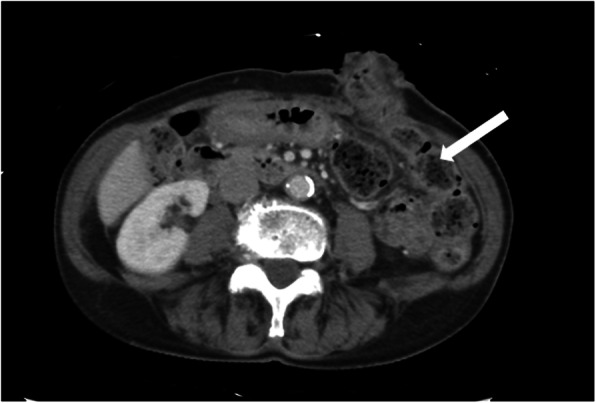
CT scan shows the lifted sigmoid colon fixed to the lateral abdominal wall

A 79-year-old woman has presented with advanced rectal cancer located on the dentate line. Computed tomography (CT) detected no metastasis. Laparoscopic APR for low rectal cancer was performed, and a sigmoid colostomy was created via the intraperitoneal route during laparoscopic APR. The histopathology of the resected tumor revealed a well-differentiated adenocarcinoma. The patient was therefore classified as T2N0M0 and Stage IIA (Fig. 1). Six months after surgery, the patient experienced vomiting, abdominal distension, and abdominal pain and visited our hospital. Contrast-enhanced CT showed the body of the stomach positioned in the lateral space between the lifted sigmoid colon and the lateral abdominal wall. In addition, the stomach was observed to be markedly dilated (Fig. 2a–c). The patient was diagnosed with internal hernia of the stomach resulting from colostomy. Initially, a nasogastric tube was inserted into the dilated stomach for drainage of gastric juice and decompression of the stomach. Then, emergency laparoscopic exploration was performed. Laparoscopy revealed that a part of the stomach had passed through a defect between the lifted sigmoid colon and left lateral abdominal wall in a cranial-to-caudal direction (Fig. 3a). The herniated stomach was already decompressed without ischemic changes. This dislocated stomach was restored to the normal position laparoscopically. The lateral defect was closed with the lateral peritoneum and the lifted sigmoid colon to avoid postoperative recurrence of IHAC (Fig. 3b). The internal hernia was repaired by interrupted suture using non-absorbable thread. There was no recurrence of IHAC after surgery (Fig. 4).

## Discussion

A colostomy is created in patients with rectal malignancies. The postoperative complications associated with colostomy are stoma stenosis, retraction, prolapse, parastomal hernia, and intestinal obstruction. Intestinal obstruction has been reported to occur in 3.5 to 7.2% of cases after stoma creation [6–8]. The major cause of intestinal obstruction is intraperitoneal adhesions. Intestinal obstruction resulting from internal hernia is a rare complication [6]. Postoperative internal hernias in many patients were caused by mesenteric defects after gastrointestinal tract or colorectal surgery [9]. A mesenteric internal hernia often leads to ischemic change or intestinal obstruction, which requires surgical treatment. Surgical closure of mesenteric defects is recommended to avoid mesenteric internal hernia. On the other hand, IHAC is rare and several cases have been reported so far [10, 11]. All the cases in previously reported literature were those of internal hernia of the small intestine. The present case is an extremely rare case of gastric obstruction by internal hernia after stoma creation. Almost all of the IHAC occurred in the small intestine, and there are no reports that the stomach is a prolapsed organ.

In this case, a part of the stomach entered into the hernia orifice as the advanced part, and the pyloric part was pressed by the lifted colon. It led to the output obstruction of the stomach, further expanding the stomach worsening the hernia. In this case, we closed the hernia orifice by fixing the lifted sigmoid colon to the abdominal wall. As a result, this treatment was successful.

Laparoscopic colorectal surgery has developed progressively in recent years, and it has many advantages compared with open surgery, including better cosmesis, lesser pain, earlier postoperative recovery, and fewer intraperitoneal adhesions. Intraperitoneal adhesions sometimes cause postoperative intestinal obstruction. Laparoscopic surgery results in fewer cases of postoperative intestinal obstruction compared with open surgery [12]. Many cases of laparoscopic colostomy are performed via the intraperitoneal route because laparoscopic colostomy creation via the extraperitoneal route is technically difficult. In contrast, conventional open surgery is performed intraperitoneally and extraperitoneally [13]. In our case and previous cases, IHAC occurred after creation of intraperitoneal colostomy. In open and laparoscopic colostomy creations, the incidence rate of IHAC was approximately 1.5%. All IHACs were observed in laparoscopic colostomy performed via the intraperitoneal route [4]. With the intraperitoneal route, the small intestine can pass through the lateral defect, and the IHAC may result in strangulation ileus and severe ischemia. Therefore, a colostomy performed by laparoscopic surgery should be created via the extraperitoneal route to prevent IHAC. Although the laparoscopic extraperitoneal colostomy technique has been developed to avoid parastomal herniation, it requires additional operating time. Moreover, if the colostomy is created through an extraperitoneal route, laparoscopic closure of the lateral space of the colostomy by suture is very difficult [14, 15]. With further developments in laparoscopic surgery, the incidence of internal hernias may increase. Adequate management of internal hernias is required, and preventive strategies for IHAC of not only the small intestine but also the stomach should be established.

## Conclusion

Internal hernia associated with colostomy can lead not only to obstruction of the small intestine, but also to obstruction of the stomach. The present case is the first report on internal hernia of the stomach associated with colostomy. During laparoscopic HO or APR, creation of colostomy via the extraperitoneal route should be considered to avoid IHAC.

## Data Availability

Not applicable

## Abbreviations

HO: Hartmann’s operation
APR: Abdominoperineal resection
IHAC: Internal hernia associated with colostomy
